# Boerhaave syndrome: an unusual myocardial infarction mimic—a case report

**DOI:** 10.1093/ehjcr/ytae310

**Published:** 2024-07-13

**Authors:** Shaunak Mangeshkar, Pawel Borkowski, Aspan Shokrekhuda, Maria Irene Barillas-Lara, Mark A Menegus

**Affiliations:** Department of Internal Medicine, Jacobi Medical Center and Albert Einstein College of Medicine, 1400 Pelham Parkway South, Bronx, NY 10461, USA; Department of Internal Medicine, Jacobi Medical Center and Albert Einstein College of Medicine, 1400 Pelham Parkway South, Bronx, NY 10461, USA; Department of Radiology, Montefiore Medical Center, Bronx, NY, USA; Division of Cardiology, Montefiore Medical Center, Bronx, NY, USA; Division of Cardiology, Montefiore Medical Center, Bronx, NY, USA

**Keywords:** Boerhaave syndrome, Myopericarditis, Myocardial infarction, Case report

## Abstract

**Background:**

A rare complication of oesophageal rupture or Boerhaave syndrome is myopericarditis due to leakage of oesophageal contents. This presentation can mimic a myocardial infarction, making diagnosis and management challenging.

**Case summary:**

We present the case of a middle-aged man presenting with chest pain, who was diagnosed with Boerhaave syndrome complicated by myopericarditis, although the presentation was concerning for acute coronary syndrome.

**Discussion:**

Through this case, we aim to highlight an unusual alternative aetiology of findings classically seen in myocardial infarction.

**Learning points**

- Our case presents an alternative explanation for findings typically seen in acute myocardial infarction, something to remember when managing patients with acute severe substernal chest pain.
- This case describes the importance of a thorough history and exam in forming a differential diagnosis of acute chest pain.
- This case emphasizes the importance of bedside cardiac ultrasonography and advanced imaging in quickly working through a differential of life-threatening diagnoses.

## Introduction

Spontaneous rupture of the oesophagus, also known as Boerhaave syndrome, is a potentially fatal condition, typically encountered in patients with severe vomiting. It classically presents with severe chest pain, and thus, the differential diagnosis of this condition may include cardiac aetiologies such as myocardial infarction (MI). A rare yet significant complication of Boerhaave syndrome is myopericarditis due to leakage of intra-oesophageal contents. Both MI and myopericarditis have a similar clinical picture with ST segment elevations on electrocardiogram (ECG) and elevated troponin levels, making the diagnosis and management challenging. We present the case of a middle-aged man diagnosed with Boerhaave syndrome complicated by myopericarditis, although the presentation was concerning for acute coronary syndrome. We aim to highlight an unusual alternative aetiology of findings classically seen in MI.

## Summary figure

**Table:** 

Day 0	Patient consumed seafood and started experiencing multiple episodes of vomiting and diarrhoea.
Day 3 (admission)	Presented to the emergency department with chest pain, shortness of breath, and weakness. Underwent emergent computed tomography imaging that revealed large left hydropneumothorax with oesophageal perforation and subsequently underwent chest tube placement.
Early on Day 4	Cardiology was consulted for ST segment elevations and elevated troponins. Findings were thought to be due to myopericarditis secondary to Boerhaave syndrome and not an acute coronary syndrome. Underwent surgical correction of oesophageal perforation.

## Case presentation

A 52-year-old male with no pertinent medical history presented to the emergency department with 3 days of chest pain, shortness of breath, and profound weakness. The patient ate seafood 3 days prior, after which he experienced several bouts of forceful vomiting and an episode of diarrhoea. Thereafter, he noticed insidious onset of chest pain and shortness of breath accompanied by fatigue and back and upper abdominal pain, which significantly worsened until presentation.

**Figure 1:**
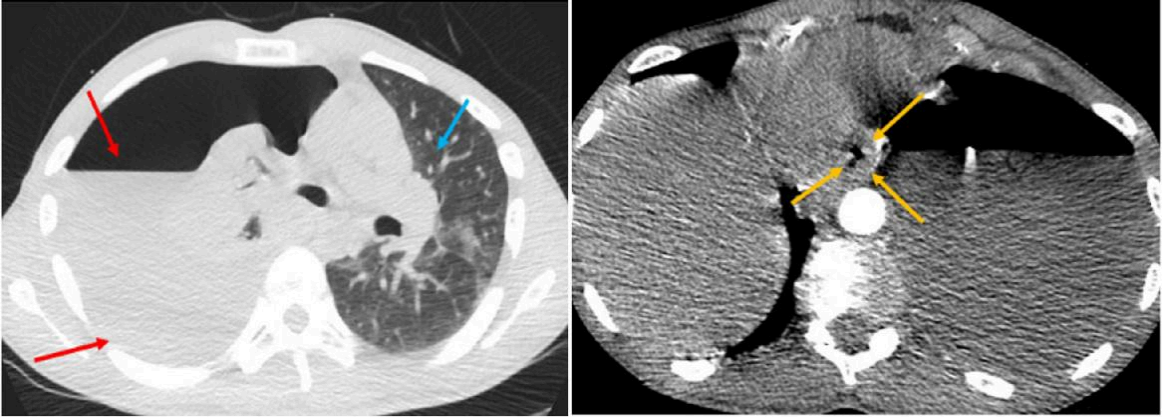
Computed tomography angiogram of the chest (lung and mediastinal window—axial computed tomography). Large left-sided hydropneumothorax (red arrows), oesophageal thickening (yellow arrows), and mediastinal shift to the right (blue arrow).

**Figure 2:**
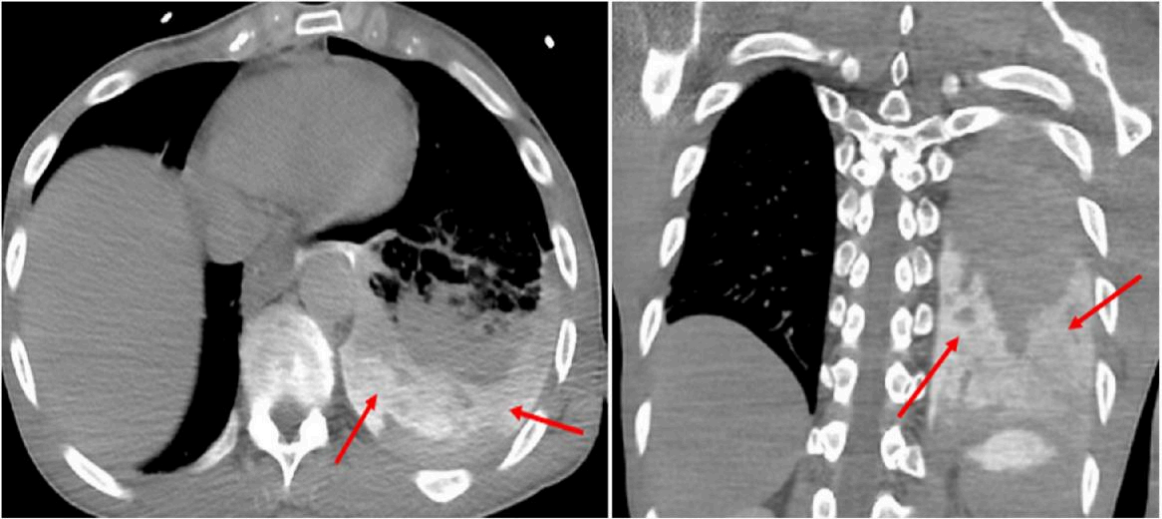
Chest computed tomography with oral contrast (axial and coronal computed tomography). Oral contrast within the left pleural space, consistent with oesophageal perforation (red arrows).

In the emergency department, the patient was noted to be severely tachypnoeic with respiratory rate nearly 40 b.p.m. and hypoxic with oxygen saturation in the range of 80–85% on room air. Cardiovascular exam was notable for a heart rate of 120 b.p.m. with a blood pressure of 120/80 mm Hg. Respiratory system examination revealed diminished breath sounds on the left-sided lower and mid-lung. There were no other pertinent physical exam findings. The patient was immediately placed on a non-rebreather mask without appreciable increase in oxygen saturation. Given the constellation of symptoms, the differential diagnosis included aortic dissection and pulmonary embolism. An emergent computed tomography (CT) angiogram of the chest, abdomen, and pelvis revealed a large left hydropneumothorax with a right mediastinal shift concerning for tension physiology along with some oesophageal thickening (*Figure 1*), but there was no evidence of pericardial effusion. A chest tube was placed emergently and drained 1600 mL of purulent fluid after which the oxygen saturation showed improvement. Intravenous fluids and antibiotics were initiated. A chest CT with oral contrast revealed leakage of contrast into the left pleural space confirming an oesophageal perforation (*Figure 2*).

**Figure 3:**
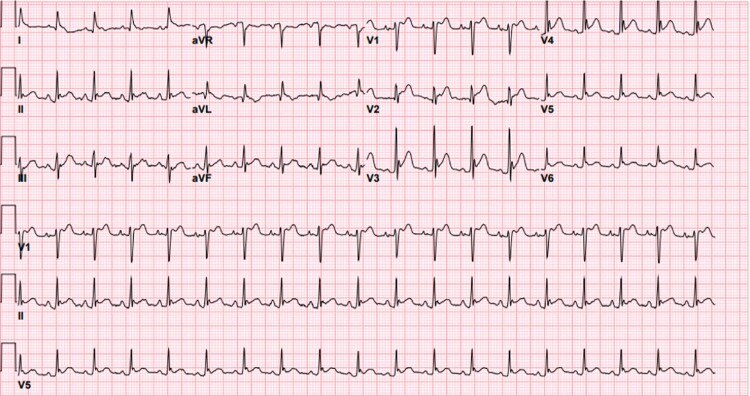
Initial electrocardiogram showing ST segment elevations, particularly demonstrable in leads V1–V5. aVR, augmented vector right; aVL, augmented vector left; aVF, augmented vector foot.

ECG showed diffuse ST segment elevations (*Figure 3*), and troponin I was also elevated with a peak of 0.41 ng/mL (normal cut-off: 0.03 ng/mL). Cardiology was consulted for evaluation and to assess the patient’s cardiac stability to undergo surgical correction. It was deemed that the findings were less likely due to ischaemic involvement but rather could be explained by inflammation of the myo-pericardium secondary to leakage of oesophageal contents. This was further supported by the absence of coronary calcifications on the chest CT scan and lack of overt risk factors for coronary disease. A bedside echocardiogram showed normal wall motion with evidence of volume depletion (Supplementary material online, *Video S1*).

The patient was taken to the operating room and underwent an endoscopy with successful repair of the oesophageal perforation using an intercostal muscle flap with intercostal nerve cryoablation.

**Figure 4:**
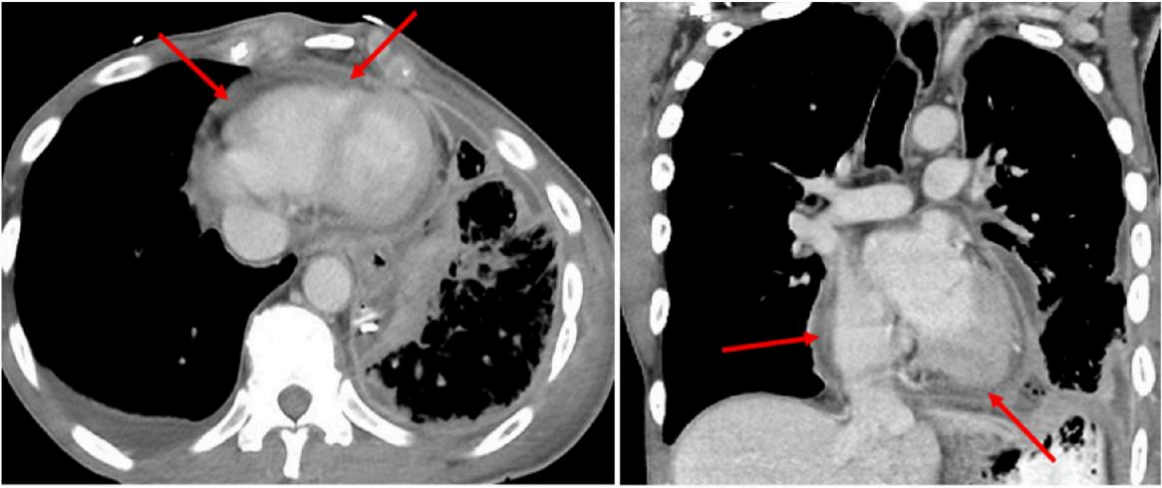
Chest computed tomography after repair of oesophageal perforation (axial and coronal computed tomography). Thickened and inflamed pericardium and pericardial fat (red arrows).

A repeated chest CT post the oesophageal perforation repair demonstrated thickened and inflamed pericardium and pericardial fat, consistent with pericarditis (*Figure 4*).

Later in the course of the hospital stay, the patient needed an oesophageal stent placement and a jejunal tube placement with eventual removal of both. The ECG at discharge showed normal sinus rhythm with resolution of previously noted ST segment elevations. On follow-up appointments, the patient was seen to be eating well, gaining weight without any further complaints. Laboratory results during follow-up showed stable blood counts and metabolic profile.

## Discussion

Chest pain is the second most common reason for adult emergency department visits in the USA, with over 7 million annual encounters.^1^ Physicians must consider life-threatening conditions, including aortic dissection, pneumothorax, acute coronary syndrome, oesophageal rupture, and pulmonary embolism, in these patients to prevent delay in diagnosis and treatment. Rapid identification of acute MI is of utmost importance in emergency medicine, given that coronary artery disease is the leading cause of death. Nevertheless, it is imperative to rule out all life-threatening causes of chest pain.

Boerhaave syndrome, with an incidence of 3.1 cases per 1 000 000 population,^2^ is a surgical emergency characterized by a spontaneous effort rupture of the oesophagus due to forceful emesis. It was originally described in 1724 by Herman Boerhaave, a Dutch Professor of Medicine, in an article pertaining to the case of an admiral presenting with vomiting followed by severe chest pain who died within 24 h. It accounts for roughly 15% of all cases of oesophageal perforation^3^ and is commonly seen in men between 50 and 70 years of age.^4^ This condition has been strongly linked to consumption of alcohol. Other risk factors include presence of gastro-oesophageal reflux disease, severe cough, and acute asthma.^5^ The mortality rate if Boerhaave syndrome is left untreated can be as high as 90%.^6^

Pathophysiologically, a rise in the intra-oesophageal pressure combined with a drop in intrathoracic pressure due to forceful vomiting leads to a transmural tear in the wall of the oesophagus, which defines Boerhaave syndrome.^7^ Most commonly, this rupture occurs in the distal third of the oesophagus and tends to involve the left lateral wall.^8^ This could be attributed to multiple anatomical considerations such as narrowing of the longitudinal muscle fibres, lack of external support, and presence of multiple vascular and nervous structures abutting the oesophagus.^9^ In most cases of oesophageal rupture, there is communication with the pleural cavity, and owing to negative intrapleural pressures, exudation of oesophageal contents into the pleural space is often seen.^5^

The presentation of a patient with Boerhaave syndrome depends on the site and extent of rupture and severity of leakage. Classically, a patient with Boerhaave syndrome presents with retrosternal chest pain usually after severe vomiting. In patients with cervical and sub-diaphragmatic perforations, they may instead present with neck pain/dysphagia and epigastric pain, respectively, instead of the classical presentation of chest pain.^10^ Clinical signs on examination include tachypnoea, tachycardia, fever, decreased breath sounds, and Hamman sign, which is a crackling sound accompanying the heartbeat, due to the presence of air in the mediastinum.

The presentation of Boerhaave syndrome in the acute setting may be confused with that of an MI considering similar degree and location of the pain. In our patient, elevated troponin levels and an ECG demonstrating diffuse ST segment elevation raised suspicion of a concomitant MI. To help exclude a possible ischaemic event, we performed a point-of-care ultrasound, which did not reveal any wall motion abnormalities and showed preserved ventricular function. Serial troponin levels were stable without a rising trend, consistent with myocardial injury rather than infarction. Computed tomography imaging of the chest confirmed the presence of oesophageal perforation and reduced the probability of ischaemia by the absence of coronary calcification.

This phenomenon of elevated troponin levels and ECG findings mimicking an MI resulted from inflammation of the myocardium and pericardium due to leakage of oesophageal contents.

## Lead author biography


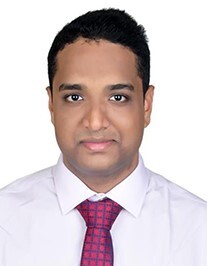


Resident Physician in Internal Medicine at Jacobi Medical Center.

## Supplementary Material

ytae310_Supplementary_Data

## Data Availability

The data underlying this article are available in the article and in its online Supplementary material.

## Notes

**Consent:** The authors confirm that informed written consent was obtained from the patient for the purpose of submission and publication of this case report in line with COPE guidelines.

**Funding:** The author(s) received no financial support for the research, authorship, and/or publication of this article.
